# Historical-crack18-19: A dataset of annotated images for non-invasive surface crack detection in historical buildings

**DOI:** 10.1016/j.dib.2022.107865

**Published:** 2022-01-24

**Authors:** Esraa Elhariri, Nashwa El-Bendary, Shereen A. Taie

**Affiliations:** a Faculty of Computers and Information, Fayoum University, Fayoum - Egypt; b College of Computing and Information Technology, Arab Academy for Science, Technology and Maritime Transport (AASTMT), Aswan - Egypt; c Faculty of Computers and Information, Misr University for Science and Technology, 6 October, Giza - Egypt

**Keywords:** Crack severity recognition, Crack, Historical buildings, Deep learning, Machine learning, Data augmentation

## Abstract

This article presents the details of Historical-crack18-19 dataset containing around 3886 annotated concrete surface images from historical buildings. The dataset comprises about 40 raw images collected from an ancient mosque (Masjid) in Historic Cairo, Egypt, with about 757 cracked and 3139 non-cracked surface instances. The images of Historical-crack18-19 dataset were captured using Canon EOS REBEL T3i digital camera with 5184 × 3456 resolution over two years (2018 and 2019). The images of Historical-crack18-19 dataset are annotated with the help of an expert and are intended for training and validation of automated non-invasive crack detection and crack severity recognition as well as crack segmentation approaches based on Machine learning (ML) and Deep Learning (DL) models. According to the environmental circumstances, where the dataset was collected, several challenges are encountered by crack detection/segmentation systems in surface images of historical buildings (illumination, crack-like patterns, separators, dust, blurring, deep texture, etc.). Further, researchers can use the dataset for benchmarking the performance of state-of-the-art methods designed for solving related (image classification and object detection problems. Historical-crack18-19 dataset is freely available at [https://data.mendeley.com/datasets/xfk99kpmj9/1].

## Specifications Table

**Table:** 

Subject	Computer Science, Artificial Intelligence, Computer Vision and Pattern Recognition
Specific subject area	Historical buildings crack detection, segmentation and severity recognition, image classification, Structural health monitoring (SHM), deep learning, Machine Learning, Surface Crack, Historical Buildings
Type of data	2D RGB images (.jpg)
How the data were acquired	Real images of cracking and non-cracking surfaces are collected from an ancient Mosque suffering from crack problems. A primary dataset about total of 40 raw images was captured using Canon EOS REBEL T3i camera with 5184 × 3456 pixels resolution over two years (2018 and 2019).
Data format	Raw
Description of data collection	40 images of cracked and non-cracked concrete segmented into more than 3886 sub- images (256 × 256 pixels). The dataset includes both narrow and wide. Many challenges facing historical buildings crack detection segmentation and severity recognition in real-world environments like illumination, crack-like, separators, dust, blurring, and deep texture, etc.
Data source location	The Mosque (Masjed) of Amir Al-Maridani (dating from 1340), located in Cairo- Egypt, with location coordinates [30.03974 N 31.25922 E].
Data accessibility	Repository name: Mendeley Data Data identification number: 10.17632/xfk99kpmj9.1 Direct URL to data: https://data.mendeley.com/datasets/xfk99kpmj9/1.
Related research article	- E. Elhariri, N. El-Bendary, S. A. Taie. 2019. Performance Analysis of Using Feature Fusion for Crack Detection in Images of Historical Buildings. In Proceedings of the 11th International Conference on Management of Digital EcoSystems (MEDES ’19). Association for Computing Machinery, New York, NY, USA, 308–315. doi:https://doi.org/10.1145/3297662.3365800 [1]. - E. Elhariri, N. El-Bendary, S. A. Taie, ``Using Hybrid Filter-Wrapper Feature Selection with Multi-Objective Improved-Salp Optimization for Crack Severity Recognition,'' in IEEE Access, 8 (2020), 84290-84315, doi:10.1109/ACCESS.2020.2991968 [2].

## Value of the Data

• Historical-Crack18-19 dataset could be useful for training and validation of algorithms for crack detection, severity recognition and crack segmentation in historical buildings.
• Historical-Crack18-19 dataset can be used to develop new ML and DL architectures to enhance the efficiency of these architectures for historical building crack detection and severity recognition.
• Historical-Crack18-19 dataset provides visual tracking of cracks in surface images of historical buildings. Therefore, it enables engineers to perform architect examinations for the early identification of structural health problems.
• Supports numerous feature selection and feature extraction methods by their color scheme, textural, and shape descriptors of different cracks.
• Original historical surfaces images are taken from a broader view, so could be desirable by the architects for the depth analysis.
• Historical-Crack18-19 dataset is collected in a natural environment with inconsistent light intensity and weather. Hence, it composes challenges for the researchers to identify the crack severity with the naked eye.

## 1 Data Description

The Historical-Crack18-19 image dataset contains about 3886 annotated images of cracked and intact walls of the historical building. Its purpose is for autonomous crack detection, severity recognition, and segmentation algorithms training, validation, and benchmarking based on computer vision, machine learning, deep convolutional neural networks (DCNN), or other techniques. Such techniques are currently widely used in the structural health monitoring field. For valuable historical buildings, continued advancement of crack detection, its severity recognition, and segmentation algorithms needs an annotated varied image dataset, which has not been available until now. Real images of historical building cracks were captured from an ancient building suffering from cracking problem (the Mosque (Masjed) of Amir Al-Maridani, located in Sekat Al Werdani, El-Darb El-Ahmar, in Cairo Governorate). It was built during the era of the Mamluk Sultanate of Cairo, Egypt in 1339-40 CE. It is distinguished by its octagonal minaret and its large dome and is considered one of the most distinctively decorated historical buildings.

This article presents the dataset containing 40 raw RGB images of cracked and non-cracked (Intact) surfaces that could help the researchers to apply various image processing and computer vision algorithms for crack detection and segmentation.

Table 1 lists the number of intact and cracked walls included in Historical-Crack18-19.

**Table 1:** Dataset description.

Historical-Crack18-19	

757 crack images	3139 intact images

**Fig. 1:**
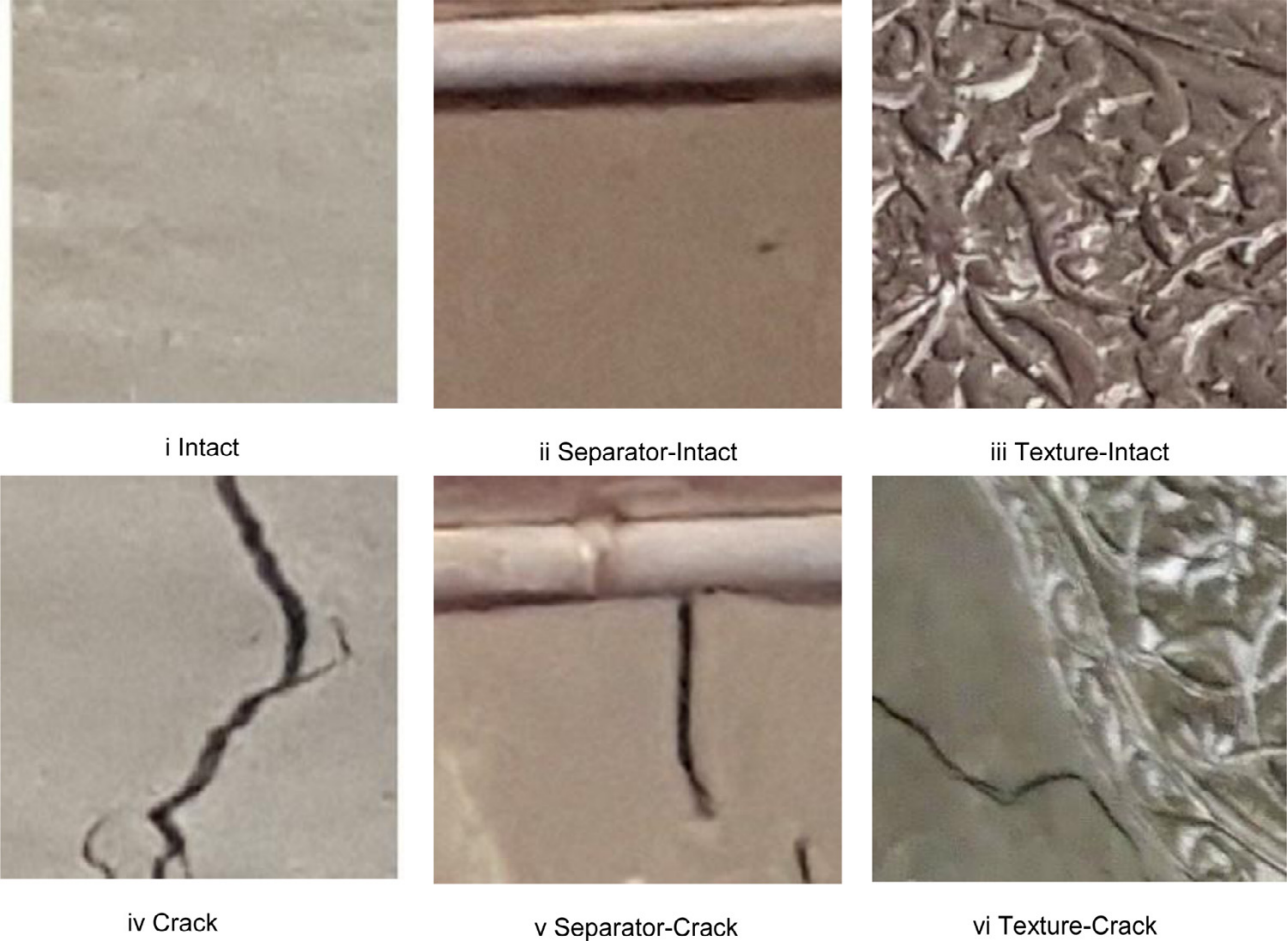
Historical-crack18-19.

**Fig. 2:**
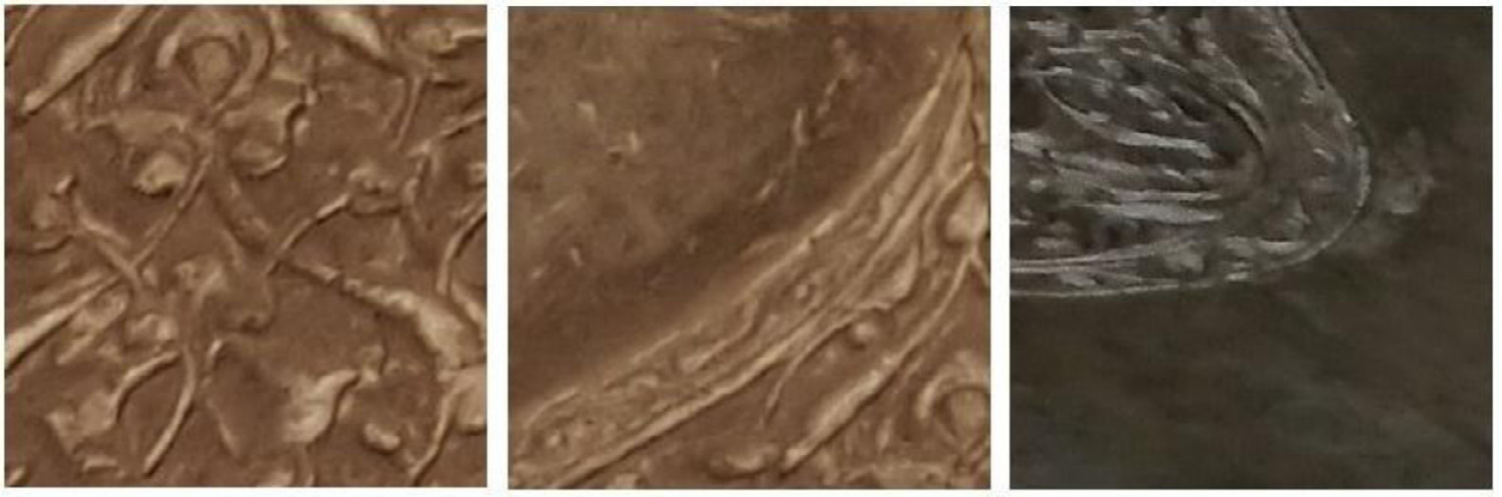
Examples of images with deep texture.

Fig. 1 show some examples from Historical-Crack18-19. Some examples of images with deep texture are shown in Fig. 2.

## 2 Experimental Design, Materials and Methods

### 2.1 Camera Specification

The collected dataset was captured using Canon EOS REBEL T3i, an advanced DSLR camera with a CMOS sensor system and resolution of 5184 × 3456 pixels. The sensor is of size 22.3 × 14.9 mm, a diagonal of 26.82 mm (1.06″) and, a surface area of 332.27 mm^2^.

### 2.2 Processing

Generally, the performance of ML and DL techniques is strongly affected by many factors such as training dataset size, the number of features, algorithm parameters, and the nature and complexity of the studied problem in some cases. Although in many domains, such as natural imaging, many datasets providing millions of images like ImageNet are available, unfortunately, the data available to train an accurate and robust classifier is insufficient in many application areas such as cracks in historical buildings due to the nature of the studied problem [3,4]. As a result of this scarcity, classification accuracy significantly decreases, and some classification models suffer from overfitting. Thus, sufficient training datasets are prerequisites for successful classification.

To tackle this problem, the original images are divided into sub-images 256 × 256 with 96 dpi resolution. As shown in Table 1 the final Historical-Crack18-19 consisted of 3886 images, which are very limited training datasets. Dataset was divided into two classes namely; intact and cracked with 3139 and 757 images, respectively. It is noted that the training dataset size needs to be enlarged for training purposes. To achieve this, the data augmentation process is suggested to be applied (could be applied) to increase training dataset size via generating new samples similar to the training samples. The most common types of data augmentation are data augmentation based on basic image manipulations (through applying geometric or color space (photometric) transformations) and data augmentations based on deep learning (through applying feature space augmentation or GAN-based data augmentation). Some example adjustments include translating, cropping, scaling, rotating, changing brightness and contrast. Unfortunately, selecting unsuitable (the inconvenient) data augmentation methods probably lead to increasing insufficiently informative samples, which have no impact or harmful impact on the classifier's accuracy and robustness [5]. The augmentation process may include some spatial and intensity transformation such as:

1. Flip image (vertically, horizontally and, vertically + horizontally),
2. Rotate image by 90 and −90 individually,
3. Flip rotated images vertically,
4. Add noises to images such as Gaussian and salt and pepper noise,
5. Apply contrast stretching to images,
6. Finally, combine the output images of steps (4, 5 and 6) to create the final augmented dataset.

## Ethics Statements

This research and analysis did not involve the use of human subjects or animal experiments.

## CRediT Author Statement

**Esraa Elhariri:** Data Collection, Initial Filtering, Data Preparation, Annotation, Writing – original draft; **Nashwa El-Bendary:** Data Collection, Data Preparation, Annotation, Supervision, Writing – review & editing; **Shereen A. Taie:** Initial Filtering, Annotation, Supervision, Writing; (The images of Historical-crack18-19 dataset are annotated with the help of an expert.).

## Declaration of Competing Interest

The authors declare that they have no known competing financial interests or personal relationships which have, or could be perceived to have, influenced the work reported in this article.
